# The Genetic Architecture of the Chickens Dropping Moisture by Genetic Parameter Estimation and Genome-Wide Association

**DOI:** 10.3389/fgene.2020.00806

**Published:** 2020-07-29

**Authors:** Tao Zhu, Tong-Yu Zhang, Junhui Wen, Xiaoyu Zhao, Yu Chen, Yaxiong Jia, Liang Wang, Xueze Lv, Weifang Yang, Zi Guan, Zhonghua Ning, Lujiang Qu

**Affiliations:** ^1^State Key Laboratory of Animal Nutrition, Department of Animal Genetics and Breeding, National Engineering Laboratory for Animal Breeding, College of Animal Science and Technology, China Agricultural University, Beijing, China; ^2^Hebei Dawu Poultry Breeding Co., Ltd., Hebei, China; ^3^Beijing Animal Husbandry and Veterinary Station, Beijing, China; ^4^Institute of Animal Sciences, Chinese Academy of Agricultural Sciences, Beijing, China

**Keywords:** chicken, dropping moisture, genetic parameters, GWAS, *COL6A3* gene

## Abstract

Dropping moisture (DM) refers to the water content of feces. High DM in chickens could be disadvantageous to pathogen control and fecal treatment in chicken farms. DM can be affected by environment, nutrition, disease, and genetics. In the present study, significant individual differences were presented in the DM of Rhode Island Red (RIR) chicken population, indicating that genetics could contribute to DM in the chickens. Subsequently, we estimated the genetic parameters of DM and conducted a genome-wide association study (GWAS) to find the potential genomic regions related to DM. The results showed that the heritability of DM ranged from 0.25 to 0.32. Furthermore, 11 significant loci on chromosome 7 were found to be associated with DM levels by the GWAS. The SNP rs15833816 within the *COL6A3* gene was the most significant SNP related to DM. Hens carrying the G allele including GA and GG produced higher DM (*P* < 0.01) levels than those carrying the other genotype AA. Our results showed that DM is a medium-inheritable trait and that *COL6A3* could be a potential candidate gene that regulates DM level in chickens.

## Introduction

The cloaca in laying hens is a passage at the end of the digestive, urinary, and reproductive systems and includes the urethra, ejaculation canal, and anal canal. Because of the unique excretory organ of chickens, feces and urine are mixed together and excreted through the cloaca, and the moisture content of hen feces is typically higher than that of some other animals.

Excess dropping moisture (DM) can severely affect the economic benefits of the laying hen industry. First, high DM can reduce the digestibility of chicken. Next, a higher DM will cause problems for fecal cleanup and fecal composting, thus eventually increasing labor costs (Ostrander and Hart, 1964). In addition, water-like feces attract more insects and bacteria in the summer, and high-DM feces are prone to releasing NH_3_, H_2_S, and other harmful gases that threaten the biological safety of laying hens (Jongebreur and Monteny, 2001; Achiano and Giliomee, 2005).

The excessive moisture content of the layer’s feces could be caused by many factors including genetics, disease, and environment. Anatomically, a chicken’s digestive system is relatively short and less time is required to digest feed in the gastrointestinal tract. Owing to the special digestive system of chickens, the DM is common in chickens. In addition, bacteria such as *Escherichia coli* (Li et al., 2012) and Pasteurella (Thanasarasakulpong et al., 2015) and viruses such as rotavirus (Kuroki et al., 1993; Wickelgren, 2000; Lim et al., 2005; Alam et al., 2011) and Newcastle disease virus (Kapczynski et al., 2013) can cause severe diarrhea in chickens.

In this study, we analyzed DM genetically. To understand the genetics of DM in chickens, we estimated the genetic parameters of DM in a layer population, i.e., Rhode Island Red (RIR) chickens, and performed a genome-wide association study (GWAS) to identify the potential genomic regions that regulate DM in the chickens.

## Materials and Methods

### Animals and Data Collection

Our experimental RIR hens were obtained from a layer breeding company in China. All hens were raised in three-tier H-shaped single cages. In total, 2,500 hens from 112 half-sib families were used to record the DM for each chicken. Observers started to record the DM when the RIR hens were 45 weeks old. Because Salmonella Pullorum (SP) and avian leukocyte virus (ALV) are common in China, we checked for the SP antibody and ALV p27 antigen titers in our birds. We confirmed that our chickens were all free of the two pathogens.

**FIGURE 1:**
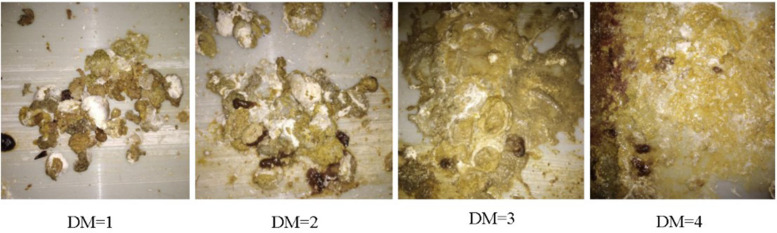
Reference diagram of DM classification in RIR hens.

Because it is difficult to detect the real water content of the feces for each of the chickens, we developed an easy and efficient method to measure the relative DM levels. By this method, the DM levels were evaluated by the appearance of the feces. The lower the DM levels, the drier were the feces. Therefore, we recorded the DM levels by numbers from 1 to 4, each corresponding to the water content of the feces, i.e., normal, slight, medium, and severe DM, respectively (Figure 1). The grading standards of our method in assessing the DM levels are shown in Table 1. The DM levels of the chickens were observed every 3 days for five times by two observers. To keep the consistency of evaluation, two observers were trained with 100 chickens until the observation results are highly consistent, and the data were reviewed during data collection.

**TABLE 1:** Classification standards for FWC of RIR hens.

**Level**	**The description of DM**
1	The dropping is spherical, with a large cluster in the middle and a small amount of granular distribution around, and almost no water in the excrement
2	Fecal irregularity, containing a small amount of undigested feed and water, most of the middle of the fecal mass
3	Feed more undigested, there is a small amount of granular, water content, fecal formless
4	The feces were completely unformed, containing undigested feed particles, and were spread almost like water on the feces scraping board

In order to study the relationship of DM to cage height and chicken performance, cage height, egg number (EN), and egg weight (EW) at age 48 weeks were recorded for further analysis.

### Genetics Parameters Estimation

**FIGURE 2:**
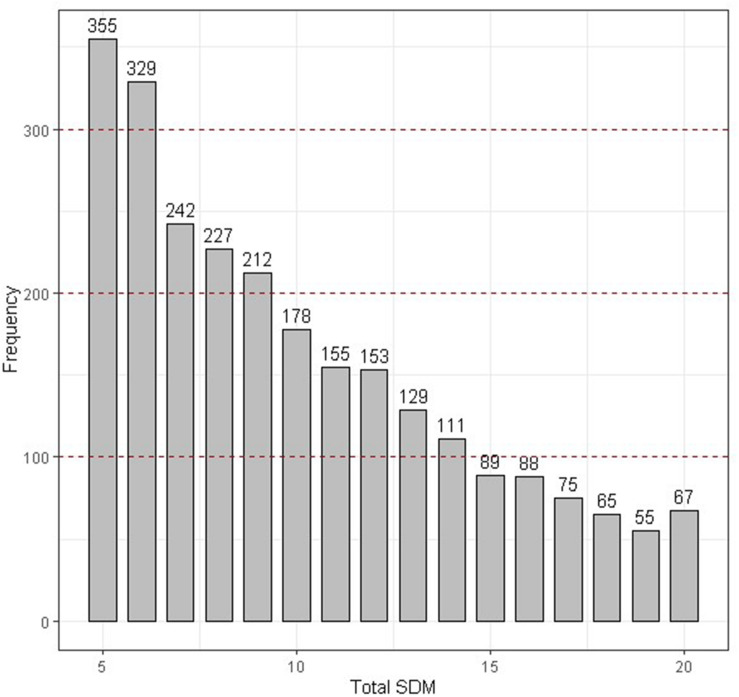
The distribution of the total SDM. The DM state was divided into four categories according to the red dotted line.

Two strategies were used in our study to estimate the genetic component variance. Strategy 1: The variance component of DM level was estimated based on the daily record; observer was included in module as a fixed variable. The variance component was estimated by Eq. 1; Strategy 2: To avoid the artificial bias, DM level numbers were added for the five times to represent the severity of DM (SDM) for each chicken, consider that SDM is not normal distribution as SDM is an integer between 5 and 20, SDM was divided into norm (SDM ≤ 6), slight (7 ≤ SDM ≤ 9), medium (10 ≤ SDM ≤ 14), and severe (SDM ≥ 15) according to the SDM distribution (Figure 2), and then the variance component was estimated Eq. 2.

(1) Y1=X1b1+Z1a1+e1

(2) Y2=X2b2+Z2a2+e2

where Y is the vector of the daily DM level; b1 represents the fixed effects including the observer and cage height; b2 is the fixed effects including the cage height; e is the vector of random residuals; [X] and [Z] are incidence matrices for the fixed and random effects, respectively. We assumed that [a]∼N([O,Aσ^2^]), [pe]∼N(0,[Iσ^2^]), and [e]∼N(0,[Iσ^2^]). The component estimation was performed using the DMU (v6) package (Madsen et al., 2006).

### Genome-Wide Association Study

We performed GWAS in order to identify the DM related genes. Individuals with SDMs of five and 20 were grouped into case and control groups, respectively. Finally, we selected 48 hens, i.e., 25 cases and 23 controls, for genome-wide genotyping. Genomic DNA was extracted from blood samples using the TIANamp Blood DNA Kit [Tiangen Biotech (Beijing) Co., Ltd.], DNA quality was tested by NanoDrop 2000 (Thermo Scientific, United States), and all the DNA samples were tested as qualified (Supplementary Table S1). Then the DNA samples were genotyped on the 600K Affymetrix Axiom chicken panel (Kranis et al., 2013), which is designed based on reference genome Gallus_gallus-5.0 and contains 580,961 SNPs with an average probe spacing of 1.79 kb. Calling and quality control was performed in the Axiom Analysis Suite; only autosomal markers with clear physical position information were used in the analysis. The subsequently quality control was performed by PLINK v1.90. SNPs were filtered based on the call rate (geno > 0.90), minor allele frequency (maf > 0.01), and Hardy–Weinberg equilibrium (hwe > 10^–5^). The samples were filtered based on the call rate (mind > 0.9). In filtration, 356,008 variants and all the 48 chicken samples passed quality control. Then, a chi-square-based genome association analysis was performed for all samples. Bonferroni correction was applied to account for multiple testing. Consider that many SNPs fall within regions of strong linkage disequilibrium (LD) (“blocks”), the suggestive and significant threshold was set at 1/LD blocks number and 0.05/LD blocks (Duggal et al., 2008). The LD blocks number was predicted by PLINK v1.90.

## Results

### DM Is a Medium-Heritable Trait

A total of 2,500 hens were phenotyped as candidate experimental individuals in our study (Table 2). According to the DMU instruction, the Gibbs sampling method was used to estimate the genetic parameter of DM. For strategy 1, the heritability of DM ranged from 0.25 to 0.31 (Table 3). For strategy 2, the heritability of DM was 0.32 (Table 3). Our results show that DM is a heritable trait with medium heritability.

**TABLE 2:** The summary of five DM records.

**Observers**	**DM state**	**31 Jul**	**3 Aug**	**6 Aug**	**9 Aug**	**12 Aug**
1	1	515	937	813	742	544
	2	255	275	264	367	390
	3	247	204	140	104	126
	4	132	106	111	118	139
2	1	269	411	421	421	611
	2	395	218	324	250	224
	3	519	212	280	234	210
	4	192	158	180	295	286
Total		2,524	2,521	2,533	2,531	2,530

**TABLE 3:** The genetic parameters of DM.

	**31 Jul**	**3 Aug**	**6 Aug**	**9 Aug**	**12 Aug**	**SDM**
Add	0.286	0.270	0.284	0.360	0.377	0.370
SE^1^	0.048	0.045	0.045	0.054	0.055	0.056
Residuals	0.733	0.775	0.758	0.783	0.812	0.769
SE^2^	0.043	0.041	0.041	0.046	0.048	0.047
h^2^	0.281	0.259	0.272	0.315	0.317	0.325

*SE^1^, stand error of additive effect.SE^2^, stand error of residuals effect.*

### DM Correlates With Cage Height

**FIGURE 3:**
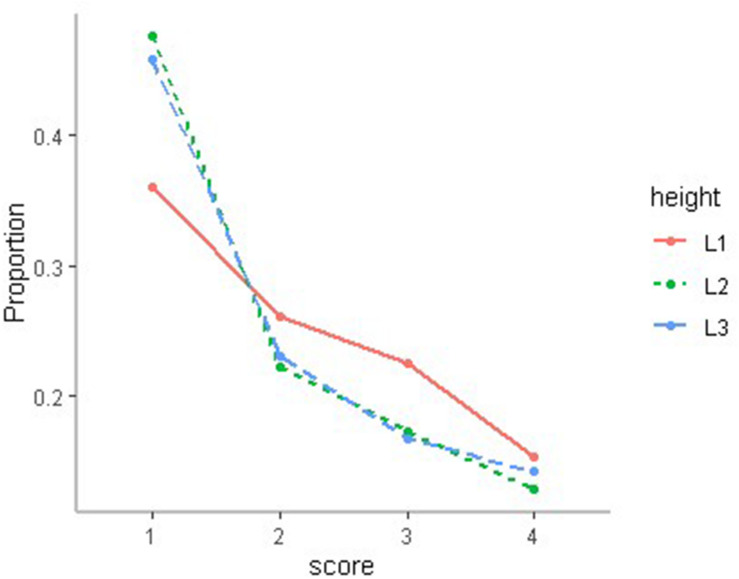
The distribution of DM state in different cages, where L1 is first floor, L2 is second floor, and L3 is third floor.

In our study, the chi-square test was used to test the effect of cage height on chicken DM, and the results showed that DM was related to cage height (*P* < 0.01). The first-level cages not only exhibited a lower proportion of slight DM but also higher proportions of middle and severe DM (Figure 3).

On the other hand, we counted the EN and randomly selected the eggs to measure the average EW at the age of 48 weeks. The EN ranged from 160 to 180, and the EW ranged from 52 to 66 g. One-way ANOVA analysis showed that EN nor EW was correlated with DM (*P* > 0.1).

### Candidate Regions Identified to Be Related to DM by GWAS

**FIGURE 4:**
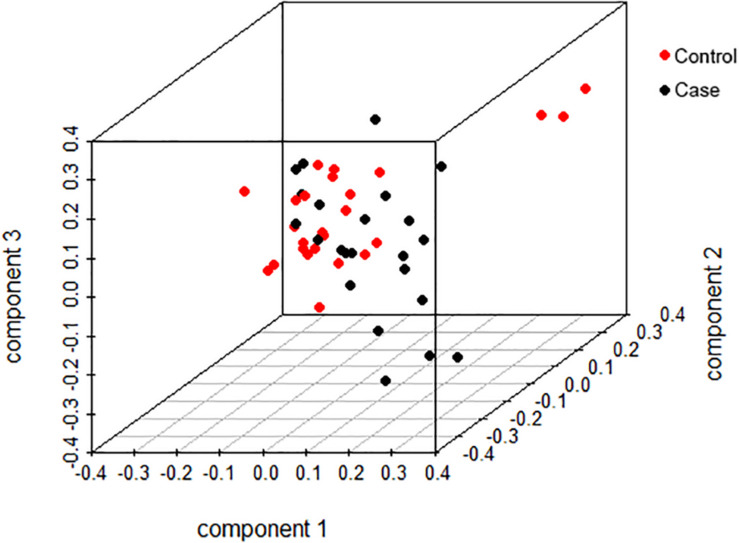
Population stratification analysis based on top the three principal components.

**FIGURE 5:**
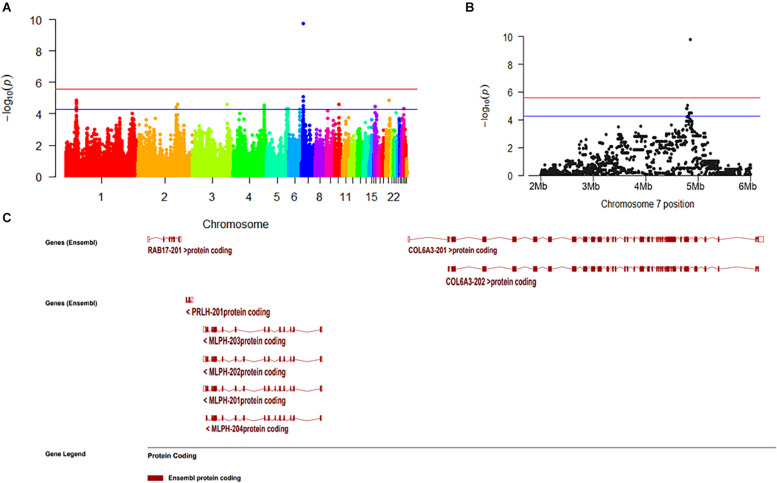
The result of genome wide association. **(A)** Manhattan plot of genome-wide association study. **(B)** Manhattan plot from 2M to 6M in chromosome 7. **(C)** The overlapping genes of significant peak on chromosome 7.

We selected 25 chickens with a total SDM of five in the case group and 23 with total SDM of 20 in the control group to perform GWA. The top-3 principal components explained more than 60% of the variance between the individuals. No significant population stratification was found in our experiment (Figure 4). A total of 18,650 LD blocks were found in the autosomes; therefore, the thresholds and significant threshold of the association analysis were set to 5.35 × 10^–5^ (1/18,650) and 2.68 × 10^–6^ (0.05/18,650), respectively. The case-control GWAS was performed for all individuals, and the results are presented in Figure 5A. Eleven regions (SNPs) were beyond the suggested line; however, only one SNP on chromosome 7 remained significant after Bonferroni correction. There is a peak around the SNP, 10 SNPs beyond the suggested line (Figure 5B). This peak overlapped with three genes, *COL6A3*, *MLPH*, *and RAB17* gene (Figure 5C). The detail SNPs information is presented in Table 4. The genotype frequencies of rs15833816 presented the most significant difference between the case and control groups, which is an intron variation of the *COL6A3* (collagen type VI alpha 3 chain) gene. Then gene ontology (GO) was employed for other autosome gene which overlap with significant SNP, but no GO item was enriched, and the gene set and SNPs set was present in Supplementary Table S2.

**TABLE 4:** SNPs significantly associated with DM in chromosome.

**Chr**	**Physical position**	**dbSNP RS ID**	**log10 (*P*-value)**	**Consequence type**	**Gene name**
7	4,849,296	rs15833816	9.76	Intron	COL6A3
7	4,800,037	rs315128343	5.06	Upstream	MLPH
7	4,771,232	rs315545865	4.80	Intron	RAB17
7	4,780,412	rs16577490	4.80	Synon	MLPH
7	4,823,591	rs15833782	4.49	Intron	COL6A
7	4,833,705	rs313522107	4.49	Intron	COL6A3
7	4,835,183	rs16577569	4.49	Intron	COL6A3
7	4,836,150	rs314895510	4.49	Synon	COL6A3
7	4,838,048	rs312605412	4.49	Synon	COL6A3
7	4,865,248	rs16577616	4.49	Downstream	COL6A3
7	4,821,683	rs14602590	4.32	Intron	COL6A3

## Discussion

### Heritability of DM

The estimation of variance component is largely dependent on the quality and quantity of data. DM level can be quantified by measuring the water content of feces of each bird. However, it was not feasible to measure the feces of each bird in our experiment. Therefore, we regarded DM as a threshold trait by assigning numbers from 1 to 4 according to the feces shape of each chicken. A similar strategy has been used in other animal studies, such as feather peaking (Brinker et al., 2014) and body condition score of cows (Edmonson et al., 1989). In the present study, the heritability in different models ranged from 0.25 to 0.32, indicating that the additive effect explained at least 25% of the total phenotypic variance. Genetic selection could be an effective method for changing the DM level in the chickens.

A previous study reported that cage height affected the behavior of hens leading to head scratching, and body shaking (Nicol, 1987; Albentosa et al., 2007). In this study, we found that cage height was strongly related to DM level. Chickens in lower cages presented higher DM levels, suggesting that sanitary conditions caused by cage height affected DM levels. There are many environmental factors decided by cage height, such as air condition, number of microorganisms, and temperature. Improving housing conditions is also an effective way to eliminate DM.

### Genome-Wide Association Study

With the development of sequencing technology and microarray technology, the cost of genome-wide genotyping is becoming lower. GWAS, an effective method to identify important molecular markers, has been widely used in animal breeding.

In our study, we identified a marker that is significantly related to DM level and three candidate regulatory genes (*CLO6A3*, *MLPH*, and *RAB17*). Considering that the most significant SNPs occurred in the *COL6A3* gene, this gene is most likely associated with DM in chickens. The *COL6A3* gene starts at 4,808,221 bp on 7 chromosome and ends at 4,861,524 bp in Gallus_gallus-5.0, and its full length is 53,303 bp. As reported in a previous study, mutations in the *COL6A3* gene can lead to poor muscle development in humans and cause Down’s syndrome (Dey et al., 2013) and muscle dystonia (Demir et al., 2002). The full name of *MLPH* gene is melanophilin, which has been found to be related to the feather color in chickens (Vaez et al., 2008). *RAB17* gene, full name is member RAS oncogene family, which is a cancer- and immunity-related gene.

## Conclusion

Dropping moisture is a medium heritable trait and a genetic marker exhibited significant association with it. Furthermore, we found that the main candidate gene *COL6A3* could affect the DM level in chickens. This provides theoretical basis for subsequent functional verification and insight into genetic selection for the DM level to help improve the economic efficiency of layer farms.

## Data Availability Statement

The datasets that support the findings of this study are available on FigShare https://figshare.com/articles/GENETICS_OF_DROPPING_MOISTURE/12027936.

## Ethics Statement

The animal study was reviewed and approved by the Committee for Animal Care and Use of China Agricultural University. Written informed consent was obtained from the owners for the participation of their animals in this study.

## Author Contributions

TZ and T-YZ performed the experiments and data analysis and wrote the manuscript. JW and ZG contributed to sample collection. XZ provided test poultry farms. YJ, YC, LW, XL, WY, and LQ designed the experiments and coordinated the study. LQ and ZN reviewed and revised the manuscript. All authors approved the submitted version.

## Conflict of Interest

XZ was employed by the company Hebei Dawu Poultry Breeding Co., Ltd. The remaining authors declare that the research was conducted in the absence of any commercial or financial relationships that could be construed as a potential conflict of interest.
